# Clinical Reports of Twenty Cases of Sterility

**Published:** 1849-11

**Authors:** R. Lee

**Affiliations:** Fellow of the Royal College of Physicians, Physician to the British Lying-in Hospital, and Lecturer on Midwifery at St. George’s Hospital


*Clinical Reports of Twenty Cases of Sterility.*—By R. Lee, M. D., F. R. S., Fellow of the Royal College of Physicians, Physician to the British Lying-in Hospital, and Lecturer on Midwifery at St. George's Hospital.

Case 1.—Mrs. M——, aged twenty-four, July 29th, 1824, was married the second time ten months ago, and had lived three years with her first husband without ever having become pregnant. Seven months ago, the catamenia became irregular, and she began to suffer from uneasiness and distention of the abdomen on the right side, and at times thought she distinctly felt the movements of an infant. She was afflicted with ovarian dropsy, and died, after having been tapped eight times. On examining the body, I found an enormous cyst, covered with coagulable lymph, filling up the abdomen, between which and the omentum, the peritoneum, and most of the pelvic viscera, there was an extensive adhesion. This large cyst was likewise connected with numerous small cysts, and the peritoneum almost everywhere was thickened and studded with tubercles of different sizes. The omentum also presented the same tuberculated appearance. The uterus was indurated, and about four times the natural size. Both ovaria were converted into hard tuberculated masses.

Case 2.—In the year 1825, in the Ukraine, I attended the Baroness F——, who was sterile, and who had likewise for some time been afflicted with what was supposed to be encysted dropsy. I drew off by tapping a large quantity of fluid, and she regained her health, and lived many years after without any return of the dropsy, but had no children.

Case 3.—July 28th, 1828, I examined the body of Mrs. N——, aged forty-three, who died of inflammation of the substance of the lungs, and bilious fever. She had been married many years, and was sterile. The uterus contained several fibro-cartilaginous tumors of different sizes, some under the peritoneum, and one, about midway between the surfaces of the uterus, of considerable dimensions. The preparation is in the museum of St. George's Hospital.

Case 4.—On the 9th of August, 1828, I examined the body of an aged woman, who had died, after nine years' suffering, from a large cyst and tumor in the hypogastrium. A trocar had been introduced, but only a small quantity of fluid escaped. There was a large fibro-cartilaginous tumor, and several small ones, found after death embedded in the walls of the uterus. The right ovarium had been converted into a tumor of similar structure, which weighed six or seven pounds ; and connected with this were several cysts, containing a clear fluid. The peritoneum was very much thickened and indurated.—Sterility had existed through life.

Case 5.—On the 21st of December, 1829, a middle-aged woman was admitted into the Middlesex Hospital in an exhausted and almost insensible state, with a large globular-shaped tumor hanging by a thick neck out of the vagina, between the thighs. Three pints of urine were accumulated in the bladder. The surface of the tumor was covered partially with coagulated blood, and it was extremely painful when touched. It was at first supposed to be the uterus inverted, and attempts were made without effect to reduce it. Afterwards, from a depression in the lower part of the tumor, like the os uteri, it was supposed to be a case of prolapsus uteri, and leeches and fomentations were applied to facilitate its reduction within the pelvis. Abdominal inflammation ensued, and she died on the 31st of December. It was reported that the patient was a married woman, but had been separated some years from her husband, and that she had led an irregular life, and had been subject to prolapsus uteri. It was not ascertained positively whether she had ever been pregnant, but it was certain that she had never carried a child to the full period. The body was examined on the 1st of January, 1830. The tumor, which still hung externally, was found to be a large polypus, attached by a thick root to the anterior part of the cervix uteri. The surface of the tumor was covered by a smooth membrane reflected over it from the mucous membrane of the uterus, with which it was continuous. The uterus was dragged low down into the vagina, but its structure was healthy. The ovaria were enlarged, and partially destroyed by inflammation.

Case 6.—In March, 1831, with Dr. Scott, of Stratton-street, I saw a lady at Mortlake, aged forty-seven, who had been married thirteen years, and had never been pregnant. The catamenia had always been regular. She had long suffered from pain, and sense of distention in the right side of the hypogastrium, and of bearing-down about the uterus. There had also been much uneasiness about the neck of the bladder, but no difficulty in passing the urine. Several years before I saw the patient, Dr. Scott had ascertained that the cavity of the pelvis was partially filled up with an irregular-shaped tumor, which adhered firmly to the back part of the uterus, and the upper part of which could be distinctly felt above the brim of the pelvis on the right side. The lips of the os uteri were healthy, but the orifice was unusually open, and the cervix was shortened, as in the advanced months of pregnancy.

Case 7.—In the month of September, 1832, I was requested by Sir Gilbert Blane to see Mrs. B——, aged sixty-two, who for many years had suffered from constant sense of weight and uneasiness in the back, loins, and hypogastrium, with almost constant purulent and sanguineous discharge from the vagina. She had been married for many years, and had never become pregnant; and from the age of forty-five, when she ceased to menstruate, she had suffered from several severe attacks of uterine hemorrhage. On examination, the hollow of the sacrum was found occupied by a large hard tumor, connected with the posterior part of the uterus. The os uteri had undergone little change; but the particular fetor of the discharge, and the constitutional symptoms, led me to suspect the existence of malignant disease of the uterus. In the course of a few months, after suffering excruciating pain in the region of the uterus, difficulty in passing the urine, with a profuse discharge of thin, offensive fluid from the vagina, several portions of small, irregular-shaped concretions escaped from the vagina, with a temporary relief of the most distressing symptoms. During the remainder of 1832, Mrs. B—— continued to suffer severely from the same symptoms, and she uniformly experienced relief after a calcareous concretion had passed from the vagina, which happened four or five times during that period. In the month of November, 1833, a few days after traveling a distance of eighty miles from the country, she was attacked with rigor, vomiting, exquisite tenderness over the lower part of the abdomen, and other symptoms of peritonitis, and died in forty-eight hours. I inspected the body the following day with Dr. Webster. The usual effects of severe peritonitis were seen on laying open the abdomen. The fundus and body of the uterus were extensively disorganized by malignant ulceration. To the posterior part of the body of the uterus was adherent a large fibro-calcareous tumor, which filled up the hollow of the sacrum, and displaced the rectum. The ulceration had extended through the parietes of the uterus to the tumor. The preparation of the parts is in the museum of St. George's Hospital.

Case 8.—June, 1831. Mrs. R——, aged forty, married fifteen years, without ever having been pregnant. Enjoyed good health until a few months ago, when a swelling appeared in the abdomen. Menstruation has continued regular till five months ago, then she became sick in the morning, with loss of appetite. There is slight edema of the feet; the symptoms did not arise from pregnancy, but the precise cause was not ascertained.

Case 9.—On the 9th of November, 1832, I saw a lady, aged fifty, who had been married in early life, and who, after giving birth to one child, twenty-six years before, became sterile. The catamenia had continued regular till she was forty-nine; leucorrhea then took place, with frequent, sharp-cutting pains across the loins and within the pelvis, in the situation of the uterus. There was weakness and numbness in the lower extremities, but no swelling. She had consulted Dr. Merriman a year and a half before, who stated that her symptoms arose from change of life, and the presence of a "substance growing in the urinary passage." The os uteri was so high up in the pelvis, that it was reached with great difficulty, and whether the uterus was sound, I could not possibly determine. The symptoms disappeared to a great degree in the course of a few months, but whether they returned I was not informed.

Case 10.—On the 23d of November, 1832, I saw a lady aged thirty-seven, who had been married in early life, and had no children. She reported that she had miscarried several times, but there were circumstances communicated by Dr. Burder, which cast a doubt upon the accuracy of this statement. A tumor had been removed from the vagina some years before, and she had afterwards suffered much from pain in the back, sense of throbbing, weight, and bearing-down in the pelvis, with frequent, almost constant, profuse discharges of various colored fluids from the vagina, sometimes like pus, at other times like water. Coagula were passed with great pain at the monthly periods, attended with sickness and faintness.—The uterus was low down in the pelvis, the orifice was smooth and healthy, but the body was very considerably enlarged. The precise cause of this enlargement could not be determined, and how the case terminated I could never learn.

Case 11.—On the 7th of December, 1832, with Dr. Davy and Mr. Palmer, I saw a case in which there was a hard irregular tumor of considerable size growing from the inside of the posterior lip of the os uteri. It was removed with a ligature; but being of a malignant nature, the patient ultimately died of cancer uteri. She was thirty-eight years of age, had long been married, and was barren.

Case 12.—Mrs. C——, aged forty-four, December 14th, 1832. In July last, began first to suffer from disorder of the bowels. After that, felt something unusual about the uterus, brought on, as she supposes, by over-walking.—Had one child twenty-two years ago; no miscarriages; no leucorrhea; great bearing-down pain on the left side of the pelvis, and pain extending down the left thigh and leg; no swelling; uterus felt large, but orifice healthy.—The probability is, that the uterus is enlarged from fibrous tumors in the walls.

Case 13.—August 21st, 1833. Mrs. N——, aged thirty, has been married twelve years, and has had no child. She has suffered at intervals, for several years, from some affection of the kidneys and bladder; the catamenia are regular, but accompanied with great pain, and, in the intervals, there is copious leucorrhea. She is subject to attacks of faintness, and is obviously very hysterical. Two years ago she consulted Sir Charles Clarke, who examined the uterus, and declared that it was perfectly healthy. I repeated the examination, and could discover no disease of the uterus.

Case 14.—On the 11th of December, 1833, at the request of Dr. Burder, I saw Mrs. H——, aged twenty, who had been married eighteen months without having become pregnant, and who, I believe, has since continued sterile. The catamenia were regular, and without pain. There was profuse leucorrhea, with great irritation of the vagina, and parts around its orifice, and tenderness of the hypogastrium, and various hysterical symptoms. The os and cervix uteri were healthy.

Case 15.—On the 1st of January, 1834, I saw Mrs. T——, aged thirty, who had been married five years, and was sterile. Menstruation was regular, but painful.—There was some leucorrhea, but I could detect no disease of any kind in the uterus.

Case 16.—On the 12th June, 1834, with Dr. Scott, at Barnes, I saw a lady, aged forty, who had been married seventeen years, and had no family. For two years and a half she had complained of great irritation and swelling about the vagina, and had suffered much from an acrid leucorrheal discharge. She had consulted Sir C. Clarke, who had prescribed cupping on the sacrum. She has lately felt an unusual enlargement of the hypogastrium. Catamenia regular. "Distinct swelling in the hypogastrium. There is a distinct enlargement behind the uterus, and I think I can feel a fluctuation in it."

Case 17.—On the 20th July, 1834, I saw a patient, aged thirty, who had been married twelve months, and had not become pregnant; the contracted state of the vagina, indeed, rendered this impossible. There was a hard ring near the orifice, through which the point of the finger could not by any degree of force be passed. Mr. Jones, of Soho-square, saw this case with me, and we resolved to dilate the contracted part slowly with bougies. Threatened attacks of peritonitis repeatedly occurred after the bougies were employed, and the complete dilatation of the part was not effected till the close of November.—The sterility was not removed. The orifice and cervix uteri did not require to be dilated, as they were pervious.

Case 18.—On the 13th August, I saw a lady, aged thirty-six, at Barnes, under the care of Dr. Scott, who had been married twelve years, and was sterile. She had long suffered severely from hysteria, and for several years there had been observed an enlargement of the left side of the abdomen, which increased to a great size, and when I first saw her, it was obvious that she was laboring under ovarian dropsy. Tapping was repeatedly performed, and death took place in 1836.

Case 19.—Mrs. H——, aged twenty-eight, August 20, 1834, married eight years, never pregnant; four years ago, had an affection of the spine, and ever since has suffered severely from pain in the epigastrium and indigestion; catamenia regular, but scanty; no disease of any kind detected in the uterus.

Case 20.—Mrs. A——, aged thirty, October 9, 1834, married fourteen years; second husband; two children soon after her first marriage, none since her second. The abdomen is large, and she feels at times a fluttering sensation, like the movements of a child; menstruation irregular; at Christmas, passed three months without any appearance; since then has been regular; sickness in the morning; appetite bad; edema of the feet and ankles in the evening; has difficulty at times in retaining the urine. She has consulted an eminent accoucheur, who has expressed a doubt of her pregnancy. Three months ago, she was examined with the stethescope, by another practitioner in midwifery, declared to be pregnant, and requested to provide her nurse. The labor did not take place at the expected time, and when I examined the uterus, found it in the unimpregnated state.

